# Which Structural Interventions for Adolescent Contraceptive Use Have Been Evaluated in Low- and Middle-Income Countries?

**DOI:** 10.3390/ijerph191811715

**Published:** 2022-09-16

**Authors:** Helen Elizabeth Denise Burchett, Dylan Kneale, Sally Griffin, Málica de Melo, Joelma Joaquim Picardo, Rebecca S. French

**Affiliations:** 1 Department of Public Health, Environments & Society, Faculty of Public Health & Policy, London School of Hygiene & Tropical Medicine, London WC1E 7HT, UK; 2 EPPI-Centre, UCL Social Research Institute, University College London, London WC1H 0NR, UK; 3 International Center for Reproductive Health: Mozambique, Maputo, Mozambique

**Keywords:** contraception, family planning, adolescent, structural, upstream, intervention evaluation, cash transfer, schooling, norms, empowerment

## Abstract

Reducing adolescent childbearing is a global priority, and enabling contraceptive use is one means of achieving this. Upstream factors, e.g., gender inequalities, fertility norms, poverty, empowerment and schooling, can be major factors affecting contraceptive use. We conducted a systematic map to understand which structural adolescent contraception interventions targeting these upstream factors have been evaluated in LMICs. We searched eight academic databases plus relevant websites and a 2016 evidence gap map and screened references based on set inclusion criteria. We screened 6993 references and included 40 unique intervention evaluations, reported in 138 papers. Seventeen evaluations were reported only in grey literature. Poverty reduction/economic empowerment interventions were the most common structural intervention, followed by interventions to increase schooling (e.g., through legislation or cash transfers) and those aiming to change social norms. Half of the evaluations were RCTs. There was variation in the timing of endline outcome data collection and the outcome measures used. A range of structural interventions have been evaluated for their effect on adolescent contraceptive use/pregnancy. These interventions, and their evaluations, are heterogenous in numerous ways. Improved understandings of how structural interventions work, as well as addressing evaluation challenges, are needed to facilitate progress in enabling adolescent contraceptive use in LMICs.

## 1. Introduction

Reducing adolescent childbearing is a global priority and an indicator for Sustainable Development Goal 3, “to ensure healthy lives and promote well-being for all at all ages” [1,2]. Contraceptive use is one means of achieving this by enabling people to choose the timing of planned pregnancies, to attain the desired number of children and to allow spacing between pregnancies to improve the health status of women and their children. Whilst barriers to contraceptive use are experienced by all ages, there is evidence that this is more likely to be felt by adolescent girls and young women (hereafter referred to as adolescent girls) than older women [3]. Unmet need for contraception, when a woman who is sexually active, fecund and does not wish to conceive at that time is not currently using any modern method, is typically higher for adolescent girls aged 15–19 years compared to those aged 20–24 years in low- and middle-income countries (LMIC) [3].

To date, the focus of reviews on the effectiveness of interventions to encourage adolescent contraceptive use has typically been on the supply of contraceptives and services, and/or individual-level demand-side factors [4,5,6,7,8,9,10]. Yet we know that upstream factors, such as gender inequalities, fertility norms, poverty, girls’ empowerment and schooling, can also be major factors affecting contraceptive use [11]. Given the strong influence that these factors can have on an individual’s knowledge, attitudes and behaviours, interventions that address these issues have the potential to have a greater impact than those targeting individual-level factors alone. Structural interventions target the structural-level factors, i.e., “the physical, social, cultural, organizational, community, economic, legal, or policy aspects of the environment” (p1) that can affect health and contraceptive behaviours [12]. 

Although the importance of upstream factors has been recognised [13,14], much research has focused on evaluating interventions targeting adolescents’ knowledge, beliefs, attitudes and skills rather than structural interventions that target these wider determinants [15,16]. For example, an evidence gap map of adolescent reproductive and sexual health impact evaluations and systematic reviews by 3ie found that the most frequently evaluated intervention type was sexual health education [17].

As part of an evidence synthesis project funded by CEDIL, we conducted a systematic map to understand what types of structural adolescent contraception interventions have been evaluated in LMICs. 

## 2. Materials and Methods

Rather than duplicate the comprehensive searches and screening conducted for the evidence gap map by 3ie, mentioned above [17], we screened all the impact evaluations they included and then conducted a systematic search from 2016 to July 2020 in eight databases, using controlled and free-text terms relating to adolescence, family planning and LMICs (see Appendix A for full details of the search strategy). Due to language proficiency within the team, searches were limited to English or Portuguese language references. We limited included papers to those published in 2005 or later, since it was then that global interest in contraceptive use grew [18] as well as evaluations of structural sexual and reproductive health interventions [17]. We used the WHO’s definition of adolescence, i.e., 10–19 years [19] and the World Bank’s definition of low- or middle-income country [20]. In addition, grey literature was sought from 16 websites (see Appendix A) and reference lists from relevant systematic reviews were screened. 

Search results were downloaded into Endnote and duplicates were removed before being uploaded into EPPI-Reviewer for screening. Each reference was screened for potential inclusion on the basis of title and abstract, using pre-specified exclusion criteria to ensure relevance (see Table 1). 

An initial sub-set of references were screened by four researchers (H.B., S.G., M.M., J.J.P.) to ensure consistency of understanding and application of criteria. Once at least 80% consistency had been achieved, the remaining references were screened by individual researchers. For those included at the title/abstract screening stage, full reports were obtained and screened by two researchers (H.B. and either S.G., M.M., J.J.P. or D.K.). Where agreement could not be reached, the paper was discussed with a third researcher. 

Where an intervention evaluation had been reported in multiple papers, these were identified as linked and one paper designated the main paper, to avoid duplicate counting.

A standardised coding tool was developed by the team to capture basic information about the study and the intervention, e.g., country, intervention activities, population, study design and outcomes reported. All included studies were coded using this tool. 

## 3. Results

We screened 6993 references on title/abstract and excluded 6727, then retrieved and screened the full text of 250; we were not able to retrieve 16 references (see Appendix B for PRISMA flow diagram). 

In total, 40 intervention evaluations were included, reported in 138 papers (i.e., 98 papers were secondary or subsequent to the main included paper) (see Appendix C for table of characteristics).

The majority of interventions were evaluated in Africa (24 studies), followed by Asia (n = 8) and South America (n = 6) and the Middle East (n = 3); five studies were multi-country. Five studies were conducted in India and in Kenya, four in Malawi and three each in Mexico, Zimbabwe and Uganda.

Seventeen of the forty intervention evaluations were reported only in grey literature.

### 3.1. Aims of the Interventions

Although to be included, studies had to report pregnancy, birth or contraceptive outcomes, only half of the interventions (n = 20) aimed to increase contraceptive use or improve sexual and reproductive health (implicitly or explicitly including contraceptive use). Another eight interventions aimed to prevent HIV infection, delay early marriage or reduce sexual abuse but did not specifically focus on contraceptive use. In just under a third of studies (n = 12), the intervention had other primary aims, such as increasing participation in education, or reducing poverty.

### 3.2. Type of Structural Interventions

A range of structural interventions were evaluated, often combined with non-structural activities, such as health service provider training or mass media campaigns (see Table 2). Most involved activities that implicitly or explicitly aimed to reduce poverty or increase economic empowerment (n = 29) or aimed to encourage participation in school (n = 17). Thirteen interventions aimed to change social norms within the community.

Although we did not consider “safe space” interventions to be structural interventions themselves, half of the structural interventions (n = 20) that we included had a safe space component. Safe space groups were where girls could meet regularly, often with a mentor (typically a slightly older woman from the community), for education, training and/or recreational purposes. We considered interventions to have a safe space component if they either explicitly described themselves as such, or if they were girls-only groups which mentioned that one of their aims was to increase girls’ social/peer networks. Although many safe space interventions followed a similar format, their content as well as their frequency and duration varied, with most meeting weekly, e.g., in the Safe and Smart Savings Products for Vulnerable Adolescent Girls program [21] or several times a week, e.g., the ELA—Tanzania program [22]; in one intervention, the First Time Parents Project, participants met monthly [23]. Activities in these safe space groups could include literacy and numeracy lessons, life skills, sexual and reproductive health education, other health education (e.g., nutrition), vocational training, financial literacy, savings account activities, community development projects, sport and recreation (see Appendix B for details). Other evaluated interventions involved small group activities, but were not considered to include a safe space component as these were not described as creating “safe spaces” for adolescent girls, nor did they explicitly aim to increase their social networks, e.g., Regai Dzive Shiri [24].

Poverty reduction/economic empowerment interventions were the most common type of structural activity in the evaluations. These interventions included different activities: financial literacy training, vocational or livelihood training, the provision of conditional or unconditional cash or non-cash transfers, microfinance, the creation of savings accounts for girls or the provision of employment opportunities (see Table 3). 

Vocational and livelihoods training activities varied from those offering girls insights into potential employment options in order to raise their aspirations, e.g., in BALIKA [25], to six-month-long vocational training courses at local training institutes, followed by a micro-grant for those who completed the training and developed a business plan, as in SHAZ! [26]. 

Ten of the twelve interventions that incorporated financial literacy training delivered this through “safe space” groups, e.g., in the Ishraq program [27]. 

Cash transfers and all of the non-cash transfers were generally provided to the adolescent girls’ household rather than to the girls directly. Most cash transfers were conditional on girls’ enrolment in, or sufficient attendance at, school, e.g., the Punjab Female School Stipend Program [28]. Other cash transfers were conditional on attendance at the intervention sessions, e.g., Girl Power—Malawi [29]. Non-cash transfers included 50 kg of lentils every six months conditional on attendance at 80+% of the intervention’s meetings (Sawki [30]), a goat at the end of a two-year intervention (Berhane Hewan [31]) or cooking oil every four months, conditional on the adolescent girl remaining unmarried (Kishoree Kontha [32]). Although some interventions offered vouchers for health services (e.g., AGI-K, Marriage: No Child’s Play [33,34]), or school supplies (e.g., Berhane Hewan, Zimbabwean comprehensive school support intervention, Kenyan school subsidies and teacher training intervention [31,35,36]), these were not considered non-cash transfers as they had limited financial value. 

Seventeen studies evaluated interventions that aimed to increase schooling, either through legislative changes (e.g., extending compulsory primary school education [37] or removing schools fees, as in the Universal Primary Education Program [38]), conditional cash transfers as in the Punjab Female School Stipend Program [28], payment of school fees [39], provision of school supplies (e.g., uniforms) [35] or working with schools, parents and/or communities to support girls re-joining or remaining i, school, e.g., Marriage: No Child’s Play [33]. 

Thirteen studies explicitly aimed to change community or social norms around gender, fertility or sexual and reproductive health issues, although others may also have aimed to do so implicitly. Activities were mostly some form of community meetings and dialogue, such as “community conversations”, e.g., in Marriage—No Child’s Play [33]. Others involved community groups working through a programme, such as in Regai Dzive Shiri [24], or developing their own action plan, such as in the Ishraq pilot and scale-up [27,40].

Most interventions lasted between 18 months and 3 years, although a few were shorter, e.g., Girl Empower—Malawi [41], or longer, e.g., the Ghanaian School Scholarship Programme [39]; for some, the duration was not clear or varied, particularly those that were government cash transfer schemes, e.g., Oportunidades [42].

### 3.3. Who Was Targeted by the Intervention?

All of the interventions targeted girls, but some also targeted other participants. Aside from the 16 cash and non-cash transfers, which almost always went to the household head (the household head may have been the adolescent girl themselves, but this was rarely clearly stated), half the interventions (n = 20) focused only on girls, and half targeted boys and girls (n = 20). Fifteen interventions targeted parents, spouses or the wider community of the adolescent girls, for example, with adult–youth and adult groups in DISHA [43]. 

### 3.4. Evaluations

Twenty interventions were evaluated using randomised controlled trials (RCTs), fourteen were non-randomised and eight were natural experiments using survey data (two studies used different designs in different areas).

There was variation in the timing of endline outcome data collection, from immediately after the intervention ended, e.g., Ishraq Pilot [40], to eight years later, e.g., the Ghanaian School Scholarship Programme [39].

For the majority of interventions (n = 30), pregnancy or birth were used as outcome measures. Twenty studies measured contraceptive use and nine included other related measures, such as ideal number of children or unmet need for family planning. 

## 4. Discussion

A range of structural interventions aiming to address upstream factors have been evaluated in terms of their impact on adolescent contraceptive use and/or pregnancy/birth. Furthermore, aside from the variation in the intervention content, there is diversity in the populations targeted and settings. There is also diversity of evaluations, in terms of the study design, follow-up period and outcome measures. This heterogeneity makes synthesis or reaching a consensus about “what works” difficult. This creates challenges for policy makers and practitioners—it can be hard to judge which intervention activities would be the most feasible and effective in their specific context.

The interventions’ mechanisms of action were often unclear; for example, cash transfers could work by reducing poverty, by incentivising certain behaviours and/or by elevating the status of the person it was conditional for (i.e., the adolescent girl in this instance). Vocational training could reduce poverty by leading to employment or income-generating activities, but it could also increase autonomy, raise aspirations, reduce social isolation and build self-confidence. A better understanding of how interventions work will enable greater learning from outcome evaluations—not just to explore which activities should be incorporated, but how best they could be adapted to suit a new context. Future evaluations should explicitly test interventions’ mechanisms of action, so that we are able to judge not just whether to replicate an intervention, but how to scale it up or introduce it into a new context. Since replication of such interventions can rarely be completely faithful to the original, either in design, implementation or the effect it has in a new context, it is crucial that we understand what are the key mechanisms through which it has an effect. This will allow attention to be placed on ascertaining whether these mechanisms have been replicated, even if the intervention activities, population or setting, are different from the original evaluation. Intervention evaluations should incorporate process evaluations for this purpose, as well as to capture implementation and contextual information that could further help to understand why or how an intervention was (or was not) effective. The subsequent phase of our project aims to explore these issues, in order to develop a mid-range theory that could be operationalised in a variety of settings and with different adolescent sub-populations.

Other systematic reviews have either included both structural and non-structural interventions (e.g., [9,44,45]) or have included a broader range of outcomes than just contraception/childbearing (e.g., [17,46,47]). Other reviews have also noted the range of outcome measures and study designs used in evaluations of structural or adolescent contraception/childbearing interventions [44,47]. This map extends the evidence gap map conducted by 3ie, not only by updating it, but also by looking more in-depth at structural contraceptive interventions specifically [17].

A limitation of this map stems from the lack of consensus around what constitutes a structural intervention, as well as challenges around classifying interventions as structural or not, based on sometimes limited information in the available documentation. As such, we may have excluded interventions that others consider structural, or included some that others would not consider structural. A further limitation was that the search was limited to English and Portuguese articles. Although we did not identify any Portuguese papers, we may have missed articles in other languages, or grey literature from Portuguese or other non-English web pages. Nevertheless, we are not aware of any other review that has identified the number and range of structural interventions evaluating contraceptive/childbearing outcomes as we have. This supports our belief that a strength of our systematic approach to identifying studies is its comprehensiveness and its inclusion of grey literature from a number of sources. Others have noted the importance of this, particularly for structural interventions [45]. Finally, by omitting abortion as an outcome, we may have missed pertinent studies (however, even if it were included, data would be under-reported since abortion is illegal in many of the included countries).

## 5. Conclusions

A range of structural interventions have been evaluated for their effect on adolescent contraceptive use and pregnancy. These interventions, and their evaluations, are heterogenous in numerous ways. A better understanding of how different structural interventions work, as well as addressing the challenges of evaluating interventions, including which outcome measures are most appropriate, is needed to facilitate progress in enabling adolescent contraceptive use in LMICs.

## Figures and Tables

**Table 1:** Exclusion criteria.

Exclusion Criteria	Description of Criteria
Year Published	Exclude if published before 2005.
Country	Exclude if the intervention was NOT conducted in low- and middle-income countries, as defined by the World Bank in 2019.
Topic	Exclude if not about sexual or reproductive health.
Study design	Exclude if not an intervention evaluation.
Outcomes	Exclude if not reporting at least one of the following outcomes: - Uptake or use of modern contraception (evaluations reporting condom use only were only included if the intervention clearly stated a goal of pregnancy prevention and condoms were used for contraceptive purposes or for dual protection); - Intention/readiness to use contraception; - Desire to avoid, delay, space or limit childbearing; - Desire to use contraception; - Pregnancy/birth.
Participants	Exclude if not focused on adolescents aged 10–19 years (only include if the intervention either targeted 10–19-year-olds, or at least 50% of study sample were aged 10–19 years, or the mean or median age was 19 years or younger, or results were presented separately for this age group).
Intervention focus	Exclude if intervention does not focus on structural interventions (girls’ economic or other empowerment, school enrolment and retention, shaping norms around gender, sexual behaviour or fertility, advocacy and other interventions to reduce gender and other inequalities).

**Table 2:** Different types of structural interventions evaluated.

Type of Structural Intervention	N
Poverty reduction/economic empowerment	29
Encouraging school participation	17
Changing community social norms	13

**Table 3:** Types of poverty reduction/economic empowerment activities evaluated.

Poverty Reduction/Economic Empowerment Activity	N
Financial literacy training	14
Vocational or livelihoods training	12
Conditional cash transfer	12
Savings accounts	9
Microfinance	6
Unconditional cash transfer	5
Non-cash transfer	5
Employment or income-generating opportunities	3

**Figure A1:**
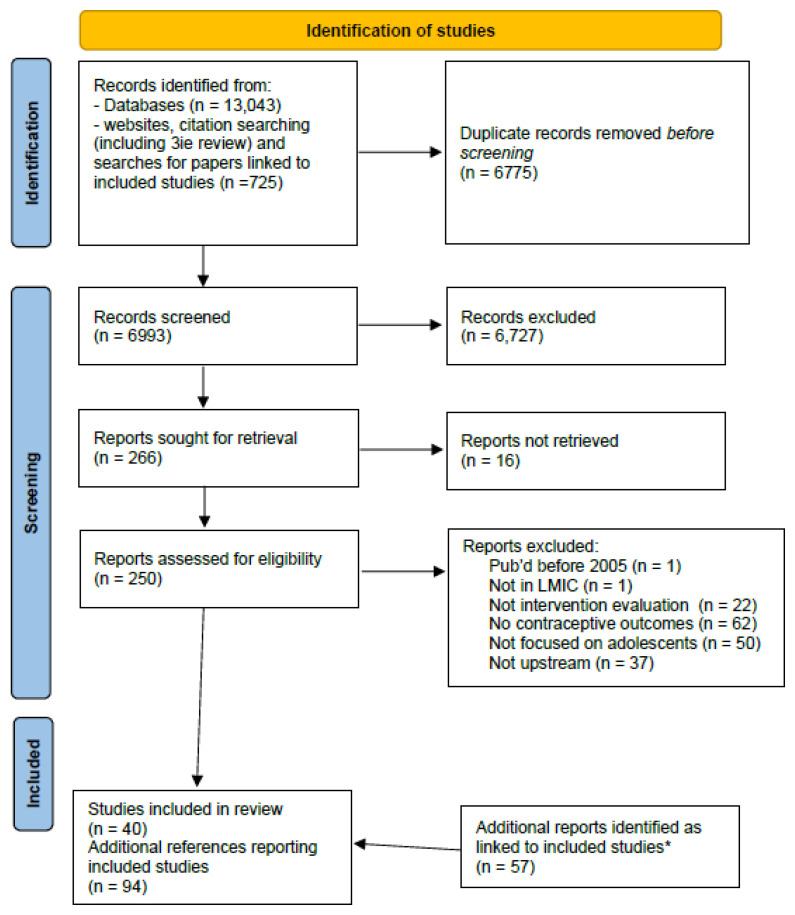
PRISMA 2020 flow diagram of references through the review.

## Data Availability

Not applicable.

## Appendix A. Search Strategy

**Databases searched**

The following bibliographic databases were searched on 29 and 30 July 2020.

- OvidSP Medline ALL, 1946 to 27 July 2020.
- OvidSP Embase, 1947 to 29 July 2020.
- OvidSP Global Health, 1910 to 2020 week 29.
- Ebsco CINAHL Plus, complete database to search date.
- Ebsco Africa-Wide Information, complete database to search date.
- Clarivate Analytics Web of Science, Science Citation Index Expanded. Year 1970–present, data last updated 16 September 2020.
- ProQuest ERIC, 1966–search date.
- WHO Global Index Medicus, complete database to search date.

**Websites hand-searched**

1. Advocates for Youth
2. Family Health International
3. Guttmacher Institute
4. Interagency Youth Working Group
5. International Center for Research on Women
6. International Planned Parenthood Federation
7. Family planning high-impact practices
8. Marie Stopes International
9. Pathfinder International
10. Population Council
11. United Nations Population Fund
12. United Nations Children’s Fund
13. World Health Organisation (WHO)
14. NBER
15. World Bank (2016 onwards)
16. JSI (2016 onwards)

**Example search strategy: Medline OvidSP**

1. adolescent/or child/ (2806512)
2. puberty/or menarche/ (17517)
3. homeless youth/ (1290)
4. minors/ (2576)
5. disabled children/ (6288)
6. students/ (58686)
7. child *.ti,ab. (1383127)
8. (girl or girls or boy or boys).ti,ab. (229162)
9. (paediatric * or pediatric *).ti,ab. (350866)
10. (schoolage * or (school adj1 age *)).ti,ab. (22762)
11. minor *.ti,ab. (295741)
12. ((school or college) adj3 (pupil * or student *)).ti,ab. (46075)
13. prepubescen *.ti,ab. (1008)
14. puberty.ti,ab. (27560)
15. pubescent *.ti,ab. (865)
16. adolescen *.ti,ab. (278039)
17. juvenil *.ti,ab. (81699)
18. underage *.ti,ab. (1211)
19. (preteen * or pre-teen *).ti,ab. (481)
20. (teen or teens or teener).ti,ab. (10684)
21. teenage *.ti,ab. (21165)
22. (youth or youths).ti,ab. (72797)
23. young people *.ti,ab. (28285)
24. young person *.ti,ab. (3499)
25. young wom#n.ti,ab. (30614)
26. (young man or young men).ti,ab. (20422)
27. (highschool or (high adj1 school *)).ti,ab. (32452)
28. sophomore *.ti,ab. (708)
29. (university adj3 student *).ti,ab. (19647)
30. (transition adj4 adult *).ti,ab. (4374)
31. emerging adult *.ti,ab. (2446)
32. young adult *.ti,ab. (94952)
33. early adult *.ti,ab. (7360)
34. freshm?n.ti,ab. (2313)
35. ((“10” or “11” or “12” or “13” or “14” or “15” or “16” or “17” or “18” or “19”) adj (year* old or year* of age)).ti,ab. (169296)
36. ((ten or eleven or twelve or thirteen or fourteen or fifteen or sixteen or seventeen or eighteen or nineteen) adj (year * old or year * of age)).ti,ab. (4540)
37. (age * adj (“10” or “11” or “12” or “13” or “14” or “15” or “16” or “17” or “18” or “19”) adj year *).ti,ab. (36798)
38. (age * adj (ten or eleven or twelve or thirteen or fourteen or fifteen or sixteen or seventeen or eighteen or nineteen) adj year *).ti,ab. (183)
39. or/1-38 (3983043)
40. exp Contraception/ (26828)
41. Family Planning Services/ (24812)
42. exp Contraceptive Devices/ (25273)
43. Contraception Behavior/ (8044)
44. family planning.ti,ab. (21238)
45. contracept *.ti,ab. (67679)
46. ((childbear * or pregnan *) adj2 (avoid * or delay * or prevent * or limit * or space or spacing or timing)).ti,ab. (9890)
47. or/40-46 (116173)
48. Developing Countries/ (74803)
49. ((developing or less * developed or under developed or underdeveloped or middle income or low * income) adj (economy or economies)).ti,ab. (561)
50. ((developing or less * developed or under developed or underdeveloped or middle income or low * income or underserved or under served or deprived or poor *) adj (countr * or nation? or population? or world)).ti,ab. (101164)
51. (low * adj (gdp or gnp or gross domestic or gross national)).ti,ab. (247)
52. (low adj3 middle adj3 countr*).ti,ab. (16855)
53. (lmic or lmics or third world or lami countr *).ti,ab. (7757)
54. transitional countr *.ti,ab. (160)
55. global south.ti,ab. (394)
56. “Democratic People’s Republic of Korea”/ (229)
57. (North Korea or (Democratic People * Republic adj2 Korea)).ti,ab. (421)
58. Cambodia/ (3310)
59. Cambodia.ti,ab. (3856)
60. Indonesia/ (10492)
61. (Indonesia or Dutch East Indies).ti,ab. (12412)
62. (Kiribati or Gilbert Islands or Phoenix Islands or Line Islands).ti,ab. (244)
63. Laos/ (1922)
64. (Laos or (Lao adj1 Democratic Republic)).ti,ab. (1966)
65. Micronesia/ (1172)
66. Micronesia.ti,ab. (656)
67. Mongolia/ (1792)
68. Mongolia.ti,ab. (4033)
69. Myanmar/ (2472)
70. (Myanmar or Burma).ti,ab. (4131)
71. Papua New Guinea/ (3453)
72. (Papua New Guinea or German New Guinea or British New Guinea or Territory of Papua).ti,ab. (4504)
73. Philippines/ (8326)
74. (Philippines or Philippine Islands).ti,ab. (8346)
75. Solomon Islands.ti,ab. (805)
76. Timor-Leste/ (204)
77. (Timor-Leste or East Timor or Portuguese Timor).ti,ab. (525)
78. Vanuatu/ (352)
79. (Vanuatu or New Hebrides).ti,ab. (690)
80. Vietnam/ (12258)
81. (Viet Nam or Vietnam or French Indochina).ti,ab. (15137)
82. American Samoa/ (183)
83. American Samoa.ti,ab. (362)
84. exp China/ (193285)
85. China.ti,ab. (180908)
86. Fiji/ (944)
87. Fiji.ti,ab. (1704)
88. Malaysia/ (15038)
89. (Malaysia or Malayan Union or Malaya).ti,ab. (16085)
90. Marshall Islands.ti,ab. (302)
91. Nauru.ti,ab. (153)
92. “Independent State of Samoa”/ (247)
93. ((Samoa not American Samoa) or Western Samoa or Navigator Islands or Samoan Islands).ti,ab. (559)
94. Thailand/ (26407)
95. (Thailand or Siam).ti,ab. (26674)
96. Tonga/ (244)
97. Tonga.ti,ab. (431)
98. (Tuvalu or Ellice Islands).ti,ab. (74)
99. Melanesia/ (1071)
100. Melanesia.ti,ab. (301)
101. Polynesia/ (1873)
102. Polynesia.ti,ab. (1298)
103. Kyrgyzstan/ (1285)
104. (Kyrgyzstan or Kyrgyz Republic or Kirghizia or Kirghiz).ti,ab. (980)
105. Moldova/ (688)
106. Moldova.ti,ab. (515)
107. Ukraine/ (15939)
108. Ukraine.ti,ab. (4675)
109. Uzbekistan/ (1895)
110. Uzbekistan.ti,ab. (1104)
111. Albania/ (839)
112. Albania.ti,ab. (1051)
113. Armenia/ (1408)
114. Armenia.ti,ab. (1044)
115. Azerbaijan/ (1202)
116. Azerbaijan.ti,ab. (1353)
117. “Republic of Belarus”/ (2064)
118. (Belarus or Byelarus or Byelorussia or Belorussia).ti,ab. (1543)
119. Bosnia-Herzegovina/ (2121)
120. (Bosnia or Herzegovina).ti,ab. (2317)
121. Bulgaria/ (6358)
122. Bulgaria.ti,ab. (4189)
123. “Georgia (Republic)”/ (1802)
124. Georgia.ti,ab. not Georgia/ (5960)
125. Kazakhstan/ (2665)
126. (Kazakhstan or Kazakh).ti,ab. (2743)
127. Kosovo/ (202)
128. Kosovo.ti,ab. (923)
129. Montenegro/ (214)
130. Montenegro.ti,ab. (823)
131. “Republic of North Macedonia”/ (557)
132. North Macedonia.ti,ab. (55)
133. Romania/ (10034)
134. Romania.ti,ab. (5512)
135. exp Russia/ (53208)
136. “Russia (Pre-1917)”/ (5981)
137. USSR/ (42765)
138. (Russia or Russian Federation or USSR or Union of Soviet Socialist Republics or Soviet Union).ti,ab. (28150)
139. Serbia/ (3133)
140. Serbia.ti,ab. (4315)
141. Turkey/ (34585)
142. (Turkey.ti,ab. not animal/) or (Anatolia or Asia Minor).ti,ab. (25104)
143. Turkmenistan/ (576)
144. Turkmenistan.ti,ab. (343)
145. Tajikistan/ (741)
146. Tajikistan.ti,ab. (580)
147. Asia, Central/ (475)
148. Asia, Northern/ (20)
149. Central Asia.ti,ab. (2269)
150. Haiti/ (3156)
151. (Haiti or Hayti).ti,ab. (3035)
152. Bolivia/ (2571)
153. Bolivia.ti,ab. (3228)
154. El Salvador/ (871)
155. El Salvador.ti,ab. (1237)
156. Honduras/ (1119)
157. Honduras.ti,ab. (1737)
158. Nicaragua/ (1480)
159. Nicaragua.ti,ab. (1852)
160. Argentina/ (15692)
161. (Argentina or Argentine Republic).ti,ab. (16531)
162. Belize/ (576)
163. (Belize or British Honduras).ti,ab. (843)
164. Brazil/ (93168)
165. Brazil.ti,ab. (82703)
166. Colombia/ (10376)
167. Colombia.ti,ab. (12026)
168. Costa Rica/ (3662)
169. Costa Rica.ti,ab. (4837)
170. Cuba/ (5016)
171. Cuba.ti,ab. (4477)
172. Dominica/ (98)
173. Dominica.ti,ab. (472)
174. Dominican Republic/ (1561)
175. Dominican Republic.ti,ab. (1887)
176. Ecuador/ (3711)
177. Ecuador.ti,ab. (4468)
178. Grenada/ (142)
179. Grenada.ti,ab. (314)
180. Guatemala/ (2966)
181. Guatemala.ti,ab. (3500)
182. Guyana/ (683)
183. (Guyana or British Guiana).ti,ab. (1080)
184. Jamaica/ (3426)
185. Jamaica.ti,ab. (3226)
186. Mexico/ (38352)
187. (Mexico or United Mexican States).ti,ab. (41958)
188. Paraguay/ (786)
189. Paraguay.mp. (1678)
190. Peru/ (8735)
191. Peru.ti,ab. (10340)
192. Saint Lucia/ (69)
193. (St Lucia or Saint Lucia or Iyonala or Hewanorra).ti,ab. (339)
194. “Saint Vincent and the Grenadines”/ (52)
195. (Saint Vincent or St Vincent or Grenadines).ti,ab. (603)
196. Suriname/ (927)
197. (Suriname or Dutch Guiana).ti,ab. (572)
198. Venezuela/ (4896)
199. Venezuela.ti,ab. (5227)
200. Djibouti/ (226)
201. (Djibouti or French Somaliland).ti,ab. (384)
202. Egypt/ (14699)
203. Egypt.ti,ab. (13915)
204. Morocco/ (5673)
205. Morocco.ti,ab. (5460)
206. Tunisia/ (8275)
207. Tunisia.mp. (10358)
208. (Gaza or West Bank or Palestine).ti,ab. (2434)
209. Algeria/ (3040)
210. Algeria.ti,ab. (3189)
211. Iran/ (26728)
212. (Iran or Persia).ti,ab. (37869)
213. Iraq/ (4619)
214. (Iraq or Mesopotamia).ti,ab. (6991)
215. Jordan/ (4207)
216. Jordan.ti,ab. (6109)
217. Lebanon/ (4260)
218. (Lebanon or Lebanese Republic).ti,ab. (4462)
219. Libya/ (1120)
220. Libya.ti,ab. (1250)
221. Syria/ (1810)
222. (Syria or Syrian Arab Republic).ti,ab. (1994)
223. Yemen/ (1381)
224. Yemen.ti,ab. (1814)
225. Afghanistan/ (3197)
226. Afghanistan.ti,ab. (5834)
227. Nepal/ (8128)
228. Nepal.ti,ab. (9629)
229. Bangladesh/ (10942)
230. Bangladesh.ti,ab. (13312)
231. Bhutan/ (458)
232. Bhutan.ti,ab. (731)
233. exp India/ (102909)
234. India.ti,ab. (97774)
235. Pakistan/ (17537)
236. Pakistan.ti,ab. (17947)
237. Maldives.ti,ab. (330)
238. Sri Lanka/ (5993)
239. (Sri Lanka or Ceylon).ti,ab. (6894)
240. Angola/ (997)
241. Angola.ti,ab. (1388)
242. Cameroon/ (5461)
243. (Cameroon or Kamerun or Cameroun).ti,ab. (6869)
244. Cape Verde/ (199)
245. (Cape Verde or Cabo Verde).ti,ab. (598)
246. Comoros/ (307)
247. (Comoros or Glorioso Islands or Mayotte).ti,ab. (554)
248. Congo/ (1848)
249. (Congo not ((Democratic Republic adj3 Congo) or congo red or crimean-congo)).ti,ab. (2549)
250. Cote d’Ivoire/ (3114)
251. (Cote d’Ivoire or Cote dIvoire or Ivory Coast).ti,ab. (3806)
252. Eswatini/ (579)
253. (eSwatini or Swaziland).ti,ab. (912)
254. Ghana/ (8167)
255. (Ghana or Gold Coast).ti,ab. (10613)
256. Kenya/ (15935)
257. (Kenya or East Africa Protectorate).ti,ab. (17819)
258. Lesotho/ (420)
259. (Lesotho or Basutoland).ti,ab. (704)
260. Mauritania/ (441)
261. Mauritania.ti,ab. (617)
262. Nigeria/ (28351)
263. Nigeria.ti,ab. (28272)
264. (Sao Tome adj2 Principe).ti,ab. (151)
265. Senegal/ (5694)
266. Senegal.ti,ab. (5639)
267. Sudan/ (4684)
268. (Sudan not South Sudan).ti,ab. (7349)
269. Zambia/ (4496)
270. (Zambia or Northern Rhodesia).ti,ab. (5215)
271. Zimbabwe/ (5793)
272. (Zimbabwe or Southern Rhodesia).ti,ab. (5620)
273. Botswana/ (1786)
274. (Botswana or Bechuanaland or Kalahari).ti,ab. (2549)
275. Equatorial Guinea/ (265)
276. (Equatorial Guinea or Spanish Guinea).ti,ab. (424)
277. Gabon/ (1449)
278. (Gabon or Gabonese Republic).ti,ab. (1722)
279. Mauritius/ (562)
280. (Mauritius or Agalega Islands).ti,ab. (967)
281. Namibia/ (1074)
282. (Namibia or German South West Africa).ti,ab. (1507)
283. South Africa/ (41839)
284. (South Africa or Cape Colony or British Bechuanaland or Boer Republics or Zululand or Transvaal or Natalia Republic or Orange Free State).ti,ab. (33743)
285. Benin/ (1539)
286. (Benin or Dahomey).ti,ab. (3401)
287. Burkina Faso/ (3219)
288. (Burkina Faso or Burkina Fasso or Upper Volta).ti,ab. (4184)
289. Burundi/ (634)
290. (Burundi or Ruanda-Urundi).ti,ab. (884)
291. Central African Republic/ (778)
292. (Central African Republic or Ubangi-Shari).ti,ab. (1014)
293. Chad/ (718)
294. Chad.ti,ab. (1153)
295. “Democratic Republic of the Congo”/ (4186)
296. (((Democratic Republic or DR) adj2 Congo) or Congo-Kinshasa or Belgian Congo or Zaire or Congo Free State).ti,ab. (4465)
297. Eritrea/ (345)
298. Eritrea.ti,ab. (536)
299. Ethiopia/ (12687)
300. (Ethiopia or Abyssinia).ti,ab. (15414)
301. Gambia/ (2407)
302. Gambia.ti,ab. (2290)
303. Guinea/ (1036)
304. (Guinea not (New Guinea or Guinea Pig* or Guinea Fowl or Guinea-Bissau or Portuguese Guinea or Equatorial Guinea)).ti,ab. (2608)
305. Guinea-Bissau/ (925)
306. (Guinea-Bissau or Portuguese Guinea).ti,ab. (1022)
307. Liberia/ (1204)
308. Liberia.ti,ab. (1541)
309. Madagascar/ (3421)
310. (Madagascar or Malagasy Republic).ti,ab. (4712)
311. Malawi/ (5263)
312. (Malawi or Nyasaland).ti,ab. (6875)
313. Mali/ (2331)
314. Mali.ti,ab. (3471)
315. Mozambique/ (2393)
316. (Mozambique or Mocambique or Portuguese East Africa).ti,ab. (3567)
317. Niger/ (1186)
318. (Niger not (Aspergillus or Peptococcus or Schizothorax or Cruciferae or Gobius or Lasius or Agelastes or Melanosuchus or radish or Parastromateus or Orius or Apergillus or Parastromateus or Stomoxys)).ti,ab. (3410)
319. Rwanda/ (2407)
320. (Rwanda or Ruanda).ti,ab. (2980)
321. Sierra Leone/ (1516)
322. (Sierra Leone or Salone).ti,ab. (2209)
323. Somalia/ (1581)
324. (Somalia or Somaliland).ti,ab. (1476)
325. South Sudan/ (149)
326. South Sudan.ti,ab. (528)
327. Tanzania/ (11298)
328. (Tanzania or Tanganyika or Zanzibar).ti,ab. (13390)
329. Togo/ (1133)
330. (Togo or Togolese Republic or Togoland).ti,ab. (1459)
331. Uganda/ (12017)
332. Uganda.ti,ab. (14085)
333. “africa south of the sahara”/ (11035)
334. africa, central/ (1278)
335. africa, eastern/ (4070)
336. africa, southern/ (2373)
337. africa, western/ (5817)
338. (“Africa South of the Sahara” or sub-Saharan Africa or subSaharan Africa).ti,ab. (21003)
339. Central Africa.ti,ab. (3108)
340. Eastern Africa.ti,ab. (975)
341. Southern Africa.ti,ab. (4279)
342. Western Africa.ti,ab. (831)
343. or/48-342 (1488989)
344. 39 and 47 and 343 (16244)
345. limit 344 to yr = “2016 -Current” (2845)
346. limit 345 to (english or portuguese) (2792)

## Appendix B

*From:* Page, M.J.; McKenzie, J.E.; Bossuyt, P.M.; Boutron, I.; Hoffmann, T.C.; Mulrow, C.D.; et al. The PRISMA 2020 statement: an updated guideline for reporting systematic reviews. *BMJ* **2021**, *372*, n71. doi: 10.1136/bmj.n71. For more information, visit: http://www.prisma-statement.org/ (accessed on 1 March 2022).

* These additional reports may have been identified through citations within included papers, or through specific searches for a named intervention evaluation. They may not have been included as a main paper if screened using the inclusion criteria, e.g., protocols or qualitative findings. However, we included them as supporting material to provide further information about included studies.

## Appendix C

**Table A1:** Characteristics of included studies.

Name (Main Reference)	Aim	Intervention Activities (FP = Family Planning; GBV = Gender-Based Violence; SRH = Sexual and Reproductive Health; SRHR = Sexual and Reproductive Health and Rights; RH = Reproductive Health; STI = Sexually Transmitted Infection; yo = Year Olds)	Population and Study Design (cRCT = cluster Randomised Controlled Trial; nRCT = non-Randomised Controlled Trial; RCT = Randomised Controlled Trial)
**Punjab Female School Stipend Program** **(Punjab FSSP)** [28] Linked references: [48]	To promote participation in public education for girls in middle school	*Intervention arm:* conditional cash transfer—conditional on 80% attendance at school *Control arm:* no cash transfer	Girls only Enrolled in grades 6–8 in public schools Pakistan Natural experiment; historical control
**Bangladeshi Association for Life Skills, Income and Knowledge for Adolescents** **(BALIKA)** [25] Linked references: [49,50,51,52]	To delay child marriage	*All intervention arms*: - Safe spaces—weekly meetings with mentor; computer and life skills - Community discussions around the importance of girls’ education and developing their skills, the risk of marrying girls early and other SRH and gender rights issues. Activities included meetings for parents/guardians, local support groups formed with community representatives, advocacy meetings, local events, district workshops Plus: *Arm 1*: educational tutoring (maths and English if in-school; computing or financial training if out-of-school) *Arm 2*: gender rights awareness training (life skills training on gender rights, negotiation, critical thinking and decision making) *Arm 3*: livelihood interventions (training in computers, entrepreneurship, mobile phone servicing, basic first aid) *Control arm*: no intervention	Girls and parents and community 12–18 yo in and out of school Bangladesh cRCT
***Mexican school legislation*** [53] No linked references	To increase schooling	*Intervention*: legislation extending compulsory schooling from 6th to 9th grade; building of schools *Control*: women not exposed to the reform (15–22 yo)	Boys and girls 6–9th grade (typically 12–14 yo) Mexico Natural experiment
**Adolescent Girls Empowerment Program (AGEP)** [34] Linked references: [54,55,56,57,58,59,60,61]	To empower adolescent girls by building their social, health and economic assets, allowing them, in turn, to reduce their vulnerabilities and capitalise on opportunities to improve their health, fertility and educational outcomes	*Arm 1*: safe spaces—weekly mentor-led girls group meetings on SRH, HIV, life skills and financial education; segmented by age and marital status *Arm 2*: arm 1 + health voucher (to use at facilities for general or SRH health services) *Arm 3*: arm 2 + provision of adolescent-friendly savings account *Control arm*: no intervention	Girls only “most vulnerable” unmarried 10–19 yo Zambia cRCT
**Safe and smart savings Products for vulnerable adolescent girls** **(Safe & Smart Savings)** [21] Linked references: [62]	Not clear but evaluation was “To understand the social, economic, and health effects of girls’ savings and safe spaces”	*Intervention arm*: - Safe spaces—weekly group meetings with mentor, stratified by age, with savings activities, health education, fun days, parent meetings - Financial education - Individual savings account with incentives to save *Control arm*: no intervention	Girls only 10–19 yo Kenya and Uganda nRCT
**Adolescent Girls Initiative-Kenya** **(AGI-K)** [63] Linked references: [64,65,66,67,68,69]	To delay childbearing for adolescent girls	*Arm 1 (control)*: “community conversations” on violence prevention and valuing girls, plus small fund for implementing action plan (structural intervention) *Arm 2*: arm 1 + conditional cash transfer for school enrolment and attendance and other education support (fees paid direct to school, kits with sanitary towels, underwear and basic school supplies, incentive paid to schools for enrolment) *Arm 3*: arm 2 + safe spaces, weekly meetings stratified by age and schooling status, with health, life skills and nutrition curriculum *Arm 4*: arm 3 + financial education, piggy bank (Wajir) or savings account (Kibera), plus small incentive (USD 3 per year)	Girls and community 11–14 yo Kenya, Wajir (rural) and Kibera (urban) RCT (Kibera) and cRCT (Wajir)
**Zomba Cash Transfer Program** **(Zomba CT)** [70] Linked references: [71,72,73,74]	HIV prevention	*Intervention arm*: conditional cash transfer for school enrolment and 80%+ attendance OR unconditional cash transfer of varying amounts for household head and individual girl *Control arm*: no intervention	Girls only 13–22 yo never married Malawi cRCT
**Empowerment and Livelihood for Adolescents** **(ELA-Uganda)** [75] Linked references: [76,77]	To break the vicious cycle between low participation in skilled jobs and high fertility	*Intervention arm*: - Life skills training - Vocational training - Safe spaces (“adolescent development clubs”), open five days a week *Control arm:* no intervention	Girls only 12–20 yo Uganda cRCT
**Empowerment & Livelihoods for Adolescents** **(ELA-Sierra Leone)** [78] Linked references: [79,80]	Young women’s socioeconomic empowerment	*Intervention:* - Safe spaces with mentor (“adolescent development clubs”), open 5× per week - Life skills training with SRH education - Vocational training (17+ yo) - Microfinance (18+ yo) *Control:* no intervention	Girls only 12–25 yo Sierra Leone, high Ebola disruption area and low Ebola disruption area cRCT
**Red de Protección Social (RPS)** [81] Linked references: [82,83]	To address current and future poverty	*Intervention*: Conditional cash transfer - Part 1 was conditional on preventive healthcare visits for U5s and attendance at health information workshops - Part 2 was conditional on school attendance and enrolment for 7–13 yo who had not yet completed 4th grade - Information sessions for adolescents on reproductive health and contraception; contraceptives available through healthcare providers *Control*: delayed intervention	Boys and girls, poor households Rural Nicaragua cRCT
**Ishraq-pilot phase (“enlightenment” or “sunrise”)** [40] Linked references: [84,85]	To transform girls’ lives	*Intervention:* - Trained program promoters (17–25 yo women), who also mentored girls - Established village committees - Safe spaces (3 h per day, 4× per week) with literacy, sports, life skills (SRHR), home and vocational skills - Health ID card - Life skills classes for 13–17 yo boys (especially participants’ brothers), to encourage gender-equitable thinking, 4× per week for six months - Workshops with parents, community leaders, youth centre staff - Parent meetings—to discuss education, reproductive health, female genital cutting *Control*: no intervention	Girls and boys and parents and community 13–15 yo Girls out of school Egypt nRCT; pre- and post-intervention with control
**Kishoree Kontha (Adolescent Girl’s Voice)** [32] Linked references: [86]	To reduce child marriage, teenage childbearing and to increase education	*Arm 1*: empowerment program - Safe spaces with peer educators, 2 h, 5–6 times per week for 6 months for curriculum, then ongoing - Education support: literacy, numeracy and oral communication - Social competency: life skills, nutritional and reproductive health knowledge - Half also received financial literacy training and encouragement to generate own income *Arm 2*: incentive—cooking oil for household every 4 months if girl remained unmarried until legal age of consent (18 yo) *Arm 3*: arm 1 + arm 2 *Control*: no intervention	Girls only 10–19 yo, arm 1 15–17 yo and unmarried, arm 2 Bangladesh cRCT
**Empowerment and Livelihood for Adolescents program (ELA–Tanzania)** [22] Linked references: [87]	To improve the human capital of young women	*Arm 1*: ELA intervention - Safe spaces (adolescent girls clubs) with mentor for recreation and socialising, five days per week with life skills training, as well as livelihood and vocational training - Community meetings with parents and village elders *Arm 2*: arm 1 + microcredit services for older girls, plus financial literacy training and business planning support *Control arm:* no intervention	Girls and parents and community 13–17 yo Tanzania cRCT
**Regai Dzive Shiri** [24] Linked references: [88,89,90]	HIV prevention—to change societal norms	*Intervention:* - Youth program for in- and out-of-school youth - Community-based program for parents and stakeholders to improve RH knowledge, parent–child communication, community support for adolescent RH - Clinic staff training to increase accessibility *Control:* delayed intervention (to 2007, year of final survey)	Girls and boys and parents and community Age unclear (“youth”) Zimbabwe cRCT
**Social Cash Transfer Program (SCTP)** **&** **Multiple Category Targeted Grant (MCTG)** [91] No linked references	SCTP: To reduce poverty and hunger, and improve school enrolment rates MCTG: To reduce extreme poverty and intergenerational transfer of poverty	*Intervention, SCTP:* unconditional cash transfer, 2 years, Malawi *Intervention, MCTG*: unconditional cash transfer, 3 years, Zambia *Control:* no intervention	Girls and boys 14–21 yo (for evaluation; programmes were for broader group of households) Most vulnerable households Malawi and Zambia cRCT
**Oportunidades** [42] Linked references: [92,93,94,95]	To reduce poverty and develop human capital in poor households via improvements in child nutrition, health and education	*Intervention:* - Cash transfer conditional on school attendance - Six monthly health check-ups for adolescents and adults - Health promotion talks to household head and students of middle–high education level - Nutritional supplementation *Control:* not exposed to intervention	Girls only 15–19 yo (for evaluation; programme available for boys and households with other ages) Mexico Natural experiment—survey of exposure to programme
***Ghanaian School scholarship programme*** [39] Linked references: [96]	To increase secondary school education	*Intervention:* four-year scholarship program for senior high school tuition fees, paid directly to school *Control:* no intervention	Boys and girls 13–25 yo Ghana RCT
***Kenyan School subsidies and teacher training*** [35] No linked references	Not explicit but assumed to encourage primary school education and HIV prevention	*Arm 1*: provision of free school uniform *Arm 2*: teaching training on HIV/AIDS prevention curriculum for upper primary school (focused on abstinence until marriage, plus discussion of condoms) (not structural) *Arm 3*: 1 and 2 *Control arm*: no intervention	Boys and girls Enrolled in 6th grade Kenya cRCT
**Shaping the Health of Adolescents in Zimbabwe (SHAZ!)** [26] Linked references: [97,98]	HIV prevention	*Intervention:* - Control arm activities - Financial literacy education - Vocational training + micro grant on completion - Integrated social support (guidance counselling plus mentors) *Control*: - RH health screening + provision of free FP every 6 months (for intervention and control groups) - Life skills education + home-based care training	Girls only 16–19 yo out-of-school orphans (lost at least 1 parent) Zimbabwe RCT
**Berhane Hewan (“Light for Eve”)** [31] Linked references: [99,100]	To reduce early marriage and support married adolescent girls	*Intervention:* - Parents of unmarried pledged that they would not be married during the 2 year programme - Goat incentive for parents, if remain unmarried and attend 80%+ of safe space meetings - Community conversations - Community water wells constructed In-school girls: - Provision of school materials, mentors to track and support attendance and performance and encouragement to remain in school Out-of-school girls: - As above, if wanted to return to school OR - Safe space groups for married (weekly) or unmarried (five times per week) girls with basic literacy and numeracy, livelihoods skills, financial literacy, group savings and loan scheme, referral to health centre for those requesting, with cost of clinic card provided *Control*: no intervention	Girls and community 10–19 yo Married and unmarried Ethiopia nRCT; pre- and post-intervention with control
***Kenyan education reform*** [37] No linked references	To increase education	*Intervention:* reform of education system—increased primary school by one year in 1985 *Control*: historical control	Girls and boys (age not stated) Kenya Natural experiment—DHS data from before/after reform
***Turkish schooling legislation*** [101] Linked reference: [102]	To increase education level	*Intervention:* - Change in compulsory schooling law—extended basic educational requirement from 5 to 8 years (free of charge) in 1997 *Control*: historical control (i.e., those aged 23+ years in 2008)	Boys and girls Turkey Natural experiment—DHS data from before/after
***Zimbabwean comprehensive school support*** [36] Linked references: [103,104,105]	HIV prevention	*Intervention:* *-* School support: fees, books, uniforms and other supplies - Female teachers trained as helpers (monitor attendance/assist with absenteeism) *Control:* no intervention	Girls only Grade 6, orphans (at least 1 parent deceased) Zimbabwe cRCT
**Mabinti Tushike Hatamu!** **(Girls Lets Be Leaders!)** [106] Linked references: [107]	To reduce vulnerability to HIV/AIDS, pregnancy and GBV	*Intervention:* - Girls’ groups with safe spaces: SRH training; financial and vocational skills; participatory action research; saving money; income generation *Control:* no intervention	Girls only 10–19 yo, out of school Tanzania nRCT; post-intervention only with control
**Cash Transfer for Orphans and Vulnerable Children** **(Kenyan Cash Transfer—OVC)** [108] Linked references: [109]	To reduce poverty	*Intervention:* unconditional cash transfer *Control*: no intervention	Boys and girls Ultra-poor households with at least one orphan/vulnerable child under 18 yo (at least one deceased parent, or parent/carer who is chronically ill) Kenya nRCT, pre- and post-intervention with control
**Child Support Grant** [110] Linked references: [111,112,113,114]	To improve the quality of life of impoverished children	*Intervention:* unconditional cash transfer *Control:* no intervention	Girls and boys Parent/caregiver of 0–18 yo, on low income South Africa Natural experiment
***Indian employment opportunities intervention*** [115] No linked references	Not explicit—assumed to increase employment	*Intervention:* employment opportunities (business process outsourcing recruiting services) *Control:* no intervention	Girls only India cRCT
**Development Initiative Supporting Healthy Adolescents** **(DISHA)** [43] Linked references: [116]	To improve SRH outcomes among youth	*Intervention:* - Established youth groups and youth resource centres (with health education and safe space) - Peer educators - Livelihoods training/groups, some linked to micro savings/credit groups - Mass communication activities - Adult groups - Adult–youth partnership groups - Training health workers on youth friendly health services - Youth depot holders, including married and unmarried (FP counselling and social marketing) *Control:* no intervention	Boys and girls and parents and community 14–24 yo, married and unmarried India nRCT; pre- and post-intervention; no control reported
**Young Agent Project** [117] No linked references	To keep adolescents in school, out of work, prevent violent and risky behaviours as well as to make them community leaders in their own *Favelas (Slums)*	*Intervention:* - Cash transfer conditional on attendance at both school and after school program (recreation, health talks, trips, computing skills, job training, internship) *Control:* no intervention	Boys and girls 15–17 yo, urban low income Brazil Natural experiment; post-hoc dataset with control
**Marriage: No Child’s Play”** **(MNCP)** [33] Linked references: [118,119,120,121]	To reduce child marriage	*Intervention:* - Girls’ groups with safe spaces: life skills, SRHR information, peer support, self-defence training, vocational training, arts and sports - Supporting schools to reduce drop out - Link girls/families to social protection schemes/income-generating opportunities - Financial literacy training - Strengthening child protection systems - Outreach SRHR services - Vouchers for SRHR services - Training service providers - Community conversations - Training officials to enforce laws and implement child marriage ban policies - Advocate for policy change *Control*: no intervention	Girls and families and communities 14–24 yo Unmarried and married India, Malawi, Mali, Niger cRCT (India and Malawi) nRCT (Mali and Niger)
**Sawki** [30] Linked references: [122,123,124]	To improve adolescent girls’ nutrition before pregnancy; to delay adolescent pregnancy	*Arm 1*: control group + safe spaces with mentor, weekly meetings - Teach life skills, essential nutrition actions, risks of early marriage and early pregnancy, the importance of education, literacy - Married girls learn more about RH - 50 kg lentils every 6 months conditional on attendance at 80%+ of meetings *Arm 2:* control group + arm 1 + livelihood training + savings and loan activities *Control arm*: Sawki development food assistance program (aim to reduce chronic malnutrition among pregnant/lactating women and children under 5 yo, and to increase local availability of and household’s access to nutrition foods) - Caregiver groups and husband schools, both providing information on nutrition and health (including contraception/fertility) - Mass media and other sensitisation on food production and nutrition - Advocacy sessions for women’s groups to obtain property ownership - Practical and technical food production support (vegetables and animals) - Village saving and loan association groups supported	Girls only 10–18 yo Niger nRCT; post-intervention with control
**Community-embedded reproductive health care for adolescents** **(CERCA)** [125] Linked references: [126,127,128,129,130,131,132,133]	To improve access to, and the use of, SRH services by adolescents	*Intervention:* - Media, workshops in health centres/community centres (Nicaragua) or schools (Bolivia and Ecuador) and discussion groups with parents/grandparents - Healthcare provider training - Contraceptive supply to health centres - Media campaigns - Information event with officials Bolivia and Ecuador only: - SRH workshops and youth groups in schools Nicaragua only: - Community-level education and door-to-door outreach - Friends of Youth (mentors) *Control:* no intervention	Boys and girls and parents and community Urban youth Nicaragua, Bolivia, Ecuador cRCT (Nicaragua) nRCT; pre- and post-intervention with control (Bolivia and Ecuador)
**Universal Primary Education Program (UPE)** [38] No linked references	Not explicit—assumed to increase primary education rates	*Intervention:* national introduction of tuition-free primary education in 1976 *Control:* women born between 1956 and 1961 (i.e., aged 15–20 when intervention started)	Boys and girls Nigeria Natural experiment
**Girl Empower** [41] No linked references	To reduce sexual abuse among females in early adolescence	*Arm 1*: Girl Empower - Safe spaces, with mentors, meeting weekly, with life skills curriculum including financial literacy and RH, community action events and graduation ceremonies with community stakeholders - Monthly parents/caregivers discussion group, to gain support from parents for intervention and to support/protect girls in their communities - Monthly cash sum (USD 2) for 8 months to start savings account, plus savings book and cash box - Training for quality health and psychosocial service providers for survivors of GBV *Arm 2*: Girl Empower + - Arm 1 - Caregivers receive conditional cash transfer for each session attended by girl *Control arm:* no intervention	Girls only 13–14 yo, rural Liberia cRCT
**Promoting Change in Reproductive Behaviour of Adolescents—phase III (PRACHAR III)** [134] Linked references: [135,136,137,138,139]	To delay the age at first birth and space subsequent births by at least 3 years	*Arm 1*: small-group education on SRH and life skills for 15–19 yo unmarried boys and girls, separately (not structural) *Arm 2*: - Arm 1 - Small-group education on RH for girls, 12–14 yo - Home visits to young married women for RH/FP counselling and referrals to FP services - Small group discussion and dialogue among young married men and young married women (separately) on RH and contraception, referrals to health services - Training of providers in youth friendly health services - Training programmes and sensitisation sessions with various groups: parents, husbands, community, healthcare providers *Control arm*: no intervention	Boys and girls and family and community 12–24 yo India nRCT; post-intervention with control
**Girl Power-Malawi** [29] Linked references: [140,141,142,143,144,145,146,147]	To impact HIV and SRH health service utilisation	*Arm 1 (control)*: standard care clinic: HIV testing, FP, STI syndromic management and condoms *Arm 2*: youth-friendly clinic including wider opening times, provider training, young peer educators (not structural) *Arm 3*: arm 2 + monthly small group sessions on HIV and SRH information, healthy and unhealthy romantic relationships, financial literacy, skills, e.g., problem solving and communication, for one year *Arm 4*: arm 3 + monthly cash transfer (to participant) conditional on attending each small group session	Girls only 15–24 yo Malawi nRCT; pre- and post-intervention with control
**First-Time Parents Project** [23] Linked references: [148]	To empower married young women and improve their sexual and reproductive health	*Intervention:* - Groups for married girls, meeting 2–3 h per month, topics such as legal literacy, vocational skills, health, gender, relationships, and worked on development projects. One group set up a group savings account - Home visits by outreach workers to young women and to their husbands, providing information on sex, communication, respect, joint decision making and RH topics including family planning - Community activities, e.g., health fairs - Opportunistic interactions with mothers-in-law and senior female family members about sexual health, contraception, antenatal, delivery and postpartum care, husbands’ roles in this period - Training health service providers on needs of young married women - Training traditional birth attendants and provision of safe delivery kits - Counselling in clinics - Provision of condoms and pill through peers and clinics - Strengthened antenatal services through outreach, financial assistance when needed for antenatal care, provided postpartum home visits *Control*: no intervention	Married young women and their husbands, families and community India nRCT; pre- and post-intervention with control
**Ishraq “sunrise”—scale-up phase** [27] Linked references: [149,150]	To address the specific needs of adolescent girls in a holistic manner	*Intervention:* - Safe spaces with mentors, 3 h per day, 4× per week, with literacy, basic maths, financial literacy, life skills, sports - Savings accounts, with initial deposit (USD 15) - Orientation of parents regarding savings account - Snacks and monthly food ration conditional on regular attendance - Graduation ceremony with community - Established village committee—to inform community about program, girls’ education and gender equity - Life skills classes for boys 13–17 yo to sensitise on gender quality, civil and human rights, self-responsibility - Tutoring for girls in Arabic, English and other school subjects - Home visits to convince parents of importance of girl’s continuing education - Community mobilisation, e.g., community seminars *Control:* no intervention	Girls and boys and parents and community 11–15 yo out-of-school girls 13–17 yo boys Egypt nRCT; pre- and post-intervention (compared participants with non-participants)
**Programa de Educacion, Salud y Alimentacion (Progresa)** **Programa de Asignación Familiar—family allowance program (PRAF II)** [151] Linked references: [95,152]	*Progresa*: To reduce poverty and invest in human capital *PRAF II:* To increase human capital accumulation, through education and health, to decrease chronic poverty	*Intervention (Progresa):* - Cash transfer conditional on school attendance, visits to public health clinics and attendance at educational workshops on health and nutrition *Intervention (PRAF II):* - Two cash transfers, one conditional on school enrolment and attendance for 6–12 yo, another conditional on regular health checks for pregnant women and under 3 yo *Control:* no intervention	Chronically poor, rural households Mexico (Progresa) Honduras (PRAF II) cRCT
**Gender Roles, Equality and Transformations Project** **(GREAT)** [153] Linked references: [154,155,156,157,158]	To reduce gender-based violence and improve sexual and reproductive health outcomes	*Intervention:* - Community action cycle—community action groups - Radio drama aimed at creating discussion around gender equality, GBV and SRH - Village health team member training - Toolkit for use in existing groups, tailored to married/parenting 15–19 yo, or unmarried, nulliparous 15–19 yo, or 10–14 yo in school *Control:* no intervention	Boys and girls and community 10–19 yo: NM/NP (newly married/newly parenting 15–19 yo), OAs (older adolescents—unmarried, nulliparous 15–19 yo) - 10–14 yo in school Uganda nRCT; pre- and post-intervention with control
